# End-of-Life Management Strategies for Fe–Mn Nanocomposites Used in Arsenic Removal from Water

**DOI:** 10.3390/polym17101353

**Published:** 2025-05-15

**Authors:** Maja Vujić, Jasmina Nikić, Mirjana Vijatovic Petrovic, Đorđe Pejin, Malcolm Watson, Srđan Rončević, Jasmina Agbaba

**Affiliations:** 1 Department of Chemistry, Biochemistry and Environmental Protection, Faculty of Sciences, University of Novi Sad, Trg Dositeja Obradovi’ca 3, 21000 Novi Sad, Serbia; jasmina.nikic@dh.uns.ac.rs (J.N.); djordje.pejin@dh.uns.ac.rs (Đ.P.); malcolm.watson@dh.uns.ac.rs (M.W.); srdjan.roncevic@dh.uns.ac.rs (S.R.); jasmina.agbaba@dh.uns.ac.rs (J.A.); 2 Institute for Multidisciplinary Research, University of Belgrade, Kneza Viseslava 1, 11000 Belgrade, Serbia; mira@imsi.rs

**Keywords:** PS-FMBO, chitosan-based foams, arsenic, regeneration, reuse, solidification/stabilization, leaching, chemical stability

## Abstract

This study investigates the regeneration, reuse, stabilization, and environmental safety of Fe–Mn polymer nanocomposites for arsenic (As) removal and their environmental safety. The regeneration performance of Fe–Mn polymer nanocomposites (PS-FMBO) used in this study was assessed through batch adsorption–desorption cycles using various eluents, including NaOH, NaOH–NaCl, and NaOH–NaOCl mixtures. The results demonstrated that 0.1 M NaOH yielded the best regeneration performance, maintaining higher adsorption efficiency over multiple cycles. Stronger desorption agents caused a significant decline in removal efficiency due to possible structural degradation of the PS-FMBO nanocomposite, suggesting that aggressive desorption conditions could compromise its long-term effectiveness. The stabilization of PS-FMBO with cement and quicklime was evaluated for immobilizing As, iron (Fe), and manganese (Mn). Leaching tests indicated that the composites effectively immobilized these contaminants, with minimal leaching observed even after prolonged aging, ensuring compliance with environmental safety regulations. Furthermore, chitosan-based foams were analyzed for their chemical stability, with leaching tests confirming low concentrations of As, Fe, and Mn, even under aggressive conditions, further reinforcing the material’s safety and environmental compliance. These findings underscore the potential of PS-FMBO composites and chitosan-based foams as sustainable materials for hazardous waste management and eco-friendly construction applications. Their ability to immobilize contaminants while maintaining structural integrity highlights their practical significance in reducing environmental pollution and advancing circular economy principles.

## 1. Introduction

Arsenic contamination in drinking water is a major environmental and public health concern in Serbia, particularly in the northern provinces and regions such as Vojvodina. Groundwater studies in Central Banat and across Vojvodina have reported arsenic concentrations up to 10 times the WHO limit of 10 µg/L, posing serious health risks [1,2,3,4,5]. This contamination is primarily attributed to natural geochemical processes, exacerbated by industrial activities and inadequate wastewater management. Chronic arsenic exposure is linked to skin lesions, respiratory diseases, cardiovascular disorders, and various cancers, necessitating urgent remediation efforts. While Serbia faces a significant arsenic crisis, the issue is also widespread in other countries, particularly in South Asia, where millions are exposed to unsafe As levels [5,6].

Arsenic contamination in groundwater is a widespread public health concern, particularly in regions where natural geochemical conditions promote its mobilization. Inorganic arsenic predominantly exists in two oxidation states: arsenite (As(III)) and arsenate (As(V)). Of these, As(III) is more toxic and mobile, especially under the reducing conditions often found in deeper aquifers, where it is released through the reductive dissolution of iron and manganese oxides. This presents a significant challenge for water treatment systems, which are generally more effective at removing As(V). As a result, many conventional processes require a pre-oxidation step to convert As(III) to As(V), increasing both operational complexity and costs. Due to the variety of arsenic species, flexible treatment methods are needed to address their diverse characteristics and groundwater chemistry. Traditional removal methods, such as coagulation, ion exchange, and reverse osmosis, often involve high costs, complex implementation, and by-product disposal challenges [7,8]. Adsorption-based techniques have emerged as a more practical and cost-effective alternative, where arsenic species (As(III) and As(V)) form stable complexes with adsorbent surfaces. However, conventional Fe-based adsorbents effectively remove As(V) but struggle with As(III) due to its neutral charge at typical groundwater pH levels [9]. Advanced materials capable of addressing both arsenic species are therefore essential.

Fe–Mn binary oxide (FMBO) nanomaterials are highly effective for arsenic removal due to their dual functionality: manganese oxides catalyze the oxidation of arsenite (As(III)) into its more easily adsorbed form As(V), while iron oxides primarily adsorb arsenate (As(V)). Their high surface area, mesoporosity, and abundance of active sites enable rapid and efficient arsenic uptake from complex aqueous solutions [10]. However, the practical application of pure FMBO nanoparticles is limited by challenges such as particle aggregation, difficult recovery from treated water, and reduced reusability, particularly under continuous flow conditions. To address these limitations, FMBO has been incorporated into composite materials by immobilizing the nanoparticles onto various carriers, including granular activated carbon, graphene oxide, and both synthetic and natural polymers [11,12,13,14,15,16,17,18]. These carriers enhance dispersion, mechanical strength, and handling of the composite materials. Natural polymers such as chitosan and starch are increasingly explored as FMBO carriers due to their biodegradability and environmental friendliness [17,18]. In contrast, synthetic polymers provide superior mechanical and thermal stability, making them more suitable for long-term use in demanding water treatment applications [12]. A recent study by Nikić et al. (2024) reported the development of FMBO-based nanocomposites by coating synthetic polymer matrices, polyethylene terephthalate (PET), and polyethylene (PE) with FMBO [12]. These composites demonstrated high arsenic removal efficiency, excellent structural integrity, and consistent reusability across multiple adsorption–desorption cycles [11]. Although FMBO-based nanocomposites show great promise for efficient and reusable arsenic removal, their end-of-life management remains a critical consideration for real-world applications. As these materials accumulate contaminants and undergo repeated use, selecting appropriate strategies for their disposal, regeneration, or repurposing is essential to ensure both environmental safety and economic viability.

The end-of-life management of adsorbents is critical for minimizing environmental risks and maximizing resource efficiency. These materials, used in water and wastewater treatment for contaminant adsorption, can undergo various disposal and recovery strategies, including landfill disposal, regeneration, resource recovery, and solidification/stabilization (S/S). However, it is also possible to reuse spent adsorbents in other industrial applications, such as in the production of freeze-casted sound-absorbing porous materials, etc. [19]. The management of spent adsorbents depends on contaminant load, structural integrity, and economic viability. Landfill disposal, while a conventional method, requires strict leaching control in order to prevent secondary contamination. Regeneration through chemical desorption (e.g., acid or alkaline washing) can restore adsorption capacity, though progressive structural degradation limits long-term reuse [20,21]. In terms of FMBO nanocomposite materials, hydrometallurgical processes, such as acid leaching and selective precipitation, could enable the recovery of Fe and Mn for applications in metallurgy, pigment synthesis, and battery production. Thermal treatments, including pyrolysis and calcination, can convert spent nanocomposites into catalytically active materials for advanced oxidation. These strategies contribute to a circular economy by minimizing waste and reducing reliance on virgin raw materials. However, secondary waste generation and economic constraints associated with regeneration and recovery necessitate comprehensive techno-economic and life-cycle assessments to optimize feasibility and ensure the environmental and economic sustainability of such processes [22,23,24].

After reaching their end-of-life, the adsorbents can also be used as building material, including concrete, bricks, and geopolymers, enhancing structural properties while immobilizing contaminants by mixing them with other conventional building materials using solidification/stabilization (S/S). S/S techniques can improve the long-term stability of spent materials before disposal or future use for different building purposes [25]. In solidification, the used adsorbent particles are encapsulated in a used immobilization agent (such as cement, quicklime, fly ash, and others) or a polymeric matrix, reducing contaminant mobility. Stabilization involves chemical reactions that alter the speciation of toxic elements, preventing their release into the environment. Studies have shown that incorporating used adsorbents into concrete or geopolymer matrices can enhance mechanical properties while immobilizing hazardous metals, making S/S a viable option for waste minimization. Despite these advantages, the long-term stability of S/S immobilized contaminants remains uncertain, particularly under fluctuating environmental conditions that may induce degradation or secondary release of metals [26,27].

Alternatively, one promising direction for the reuse of spent adsorbents, such as Fe–Mn polymer nanocomposites, is in the production of porous materials for sound absorption. As energy resources become increasingly limited, the use of foam thermal insulation materials in buildings has become essential for enhancing energy efficiency. These materials contribute to reducing dependence on heating, ventilation, and air conditioning (HVAC) systems, thereby lowering energy consumption and conserving natural resources. Moreover, foam insulation improves indoor thermal comfort and provides acoustic benefits by reducing noise transmission [24]. Polymer-based foam insulation materials, derived from petrochemicals, are widely used in construction but decompose slowly and contribute to pollution [28,29]. Their high flammability further raises safety concerns in indoor applications [30]. Renewable alternatives, such as chitosan, have gained attention for their biodegradability, recyclability, and sustainability. As a biopolymer, chitosan is safe, easy to handle, and has aesthetic benefits. It is widely used in food, healthcare, textiles, and water treatment and is ideal for developing eco-friendly foams [31,32]. Chitosan, being a biodegradable and biocompatible polymer, is often utilized as a matrix material for various applications. Additionally, chitosan foam can easily encapsulate waste beads, effectively immobilizing them within the matrix, resulting in the prevention of the dispersion of waste materials and minimizing environmental contamination. A few types of polysaccharides, cellulose and chitosan, have been reported as the raw materials to fabricate freeze-casted sound-absorbing porous materials [29,33,34]. The freeze-casting technique, which creates highly porous structures through directional freezing, allows precise control over pore size, optimizing both mechanical and acoustic properties. However, freeze-drying of chitosan foams requires precise control of temperature and pressure to preserve their porous and fragile structure. During primary drying, the product temperature must remain below the collapse temperature (Tc), which is specific to chitosan formulations, to avoid shrinkage or loss of structural integrity. Heat is typically applied via shelf contact and must be carefully regulated—excessive heat can cause the formation of free water, leading to partial melting and collapse, thus altering the drying mechanism from sublimation to vacuum drying [35,36,37]. Chamber pressure governs the sublimation rate and must be optimized based on the thermal and physical properties of the chitosan foam, although no universal range exists due to formulation variability. In the secondary drying phase, temperature can be gradually increased, but must remain below the glass transition temperature (Tg) of the dried chitosan matrix to avoid pore deformation. Even minor deviations in pressure or temperature can significantly impact the drying kinetics and microstructure of chitosan foams. However, many studies fail to report or rationalize the freeze-drying parameters applied to such systems [37,38,39]. Repurposing spent Fe–Mn polymer nanocomposite adsorbents into freeze-cast sound-absorbing materials offers dual benefits: it provides an eco-friendly recycling solution and enhances sound absorption properties due to the material’s inherent porosity. Additionally, this approach, combining freeze-casting with waste reuse, offers a cost-effective, sustainable alternative for soundproofing applications.

This study aims to investigate the influence of aging (7, 28 days, and 3 months) of solidified and stabilized mixtures of Fe–Mn polymer nanocomposites contaminated with As and selected immobilization agents (cement and quicklime) and their potential use as construction material. The potential of these materials for use in construction will be assessed through two leaching tests (TCLP and DIN 39414-S4) simulating real environmental conditions, as well as sequential extraction to analyze the mobility and binding of As in the Fe–Mn polymer nanocomposite. This study also investigates the possible reuse of Fe–Mn polymer nanocomposites, after reaching their end-of-life as adsorbents, in the production of freeze-cast sound-absorbing materials. The focus will be on evaluating the acoustic properties, structural integrity, and environmental benefits of such materials.

## 2. Materials and Methods

### 2.1. Materials

Commercial granular polystyrene (PS) was purchased from Sigma Aldrich (St. Louis, MO, USA), FeSO_4_·7H_2_O (Lach-Ner) and KMnO_4_ (Across Organics, Geel, Belgium) were used for FMBO nanocomposite preparation. As_2_O_3_ and As_2_O_5_ (Sigma Aldrich) were used for the preparation of stock solutions of As(III) and As(V) by dissolving them in deionized water. For the study, the reusability of Fe–Mn polymer nanocomposites, NaOH, NaOCl, and NaCl were obtained from Sigma Aldrich. Chitosan powder (low molecular weight, deacetylated chitin, poly(D-glucosamine), and 2 M glacial acetic acid (CH_3_COOH, 99+%) used as a chitosan solvent, were also obtained from Sigma Aldrich. The PS-FMBO beads used in this study were a waste material, saturated after their use for water purification.

### 2.2. Reusability of FMBO Nanocomposite

The reusability of the FMBO polymer nanocomposite was evaluated through three successive adsorption–desorption cycles. In the adsorption phase, 0.5 g of the adsorbent was introduced into glass bottles containing 20 mL of arsenic solution (0.2 mg/L). The suspensions were stirred for 4 h at pH 7.0 ± 0.2 to ensure adequate interaction between the adsorbent and arsenic species. Following adsorption, the solid phase was separated from the solution, and the residual arsenic concentration in the supernatant was quantified using inductively coupled plasma mass spectrometry (ICP-MS). The spent adsorbent was then subjected to a gentle rinse with deionized water and subsequently dispersed in 20 mL of regenerant solution, where it was agitated for 4 h to facilitate desorption.

To determine the most efficient regeneration protocol for FMBO polymer nanocomposites, preliminary investigations were conducted to assess the effectiveness of various regeneration solutions, including 0.1 M NaOH, 0.5 M NaOH, and a ternary mixture of NaOH, NaOCl, and NaCl. Conventional regeneration of FMBO-based adsorbents typically employs a combination of NaOH, NaOCl, and NaCl to enhance arsenic desorption and promote the oxidative conversion of Mn(II) to Mn(IV). However, experimental findings in this study demonstrated that 0.1 M NaOH alone was sufficient for effective arsenic desorption, thereby offering a simplified and more efficient regeneration strategy for the tested materials. Consequently, 0.1 M NaOH was selected as the optimal regenerant for the FMBO polymer nanocomposite. The extent of arsenic desorption was determined by analyzing the arsenic concentration in the regenerant solution. Before initiating the next adsorption–desorption cycle, the adsorbent was thoroughly rinsed with deionized water until the pH stabilized to a neutral pH 7.

### 2.3. Immobilization Agents and PS-FMBO Preparation for Stabilization Treatment

In order to immobilize arsenic within the contaminated FMBO polymer nanocomposite, cement (C) and quicklime (L) were utilized as stabilizing agents in the solidification/stabilization (S/S) process. The composition of the immobilization matrices applied in this treatment is provided in Table 1.

**Figure 1:**
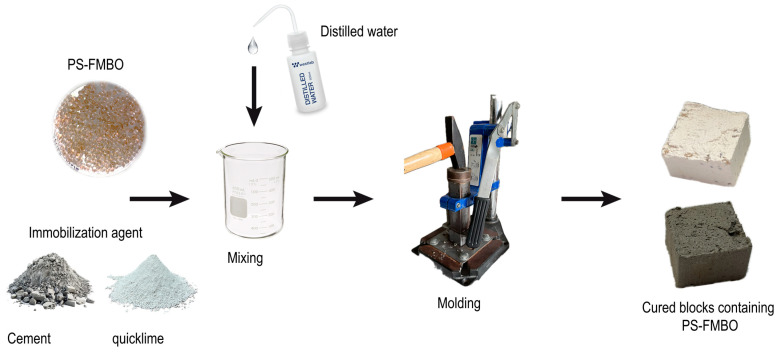
Procedure for the preparation of the stabilized and solidified PS-FMBO matrix.

The prepared mixtures were subjected to an aging process for 7 days, 28 days, and 3 months to systematically evaluate the effects of long-term curing on the mobility of arsenic, iron, and manganese in the stabilized and solidified PS-FMBO matrices. The preparation of the S/S mixtures involved the addition of the optimal water content, followed by thorough homogenization and compaction in accordance with ASTM D1557–00 [40]. A diagram of the preparation of the stabilized and solidified PS-FMBO matrix is given in Figure 1.

Following preparation, sealed plastic containers of the mixtures underwent controlled curing at room temperature for 7, 28, and 3 months. In order to observe the hydration and pozzolanic reactions during curing, specific time intervals were chosen to capture the distinct physicochemical changes. Structural development starts in the 7-day curing period, while chemical equilibrium is achieved by day 28. A 3-month aging period was chosen to evaluate the long-term stabilization of arsenic, iron, and manganese and their potential leaching. Following the S/S remediation, standardized leaching tests determined its effectiveness.

### 2.4. Preparation of Chitosan Foams

**Figure 2:**
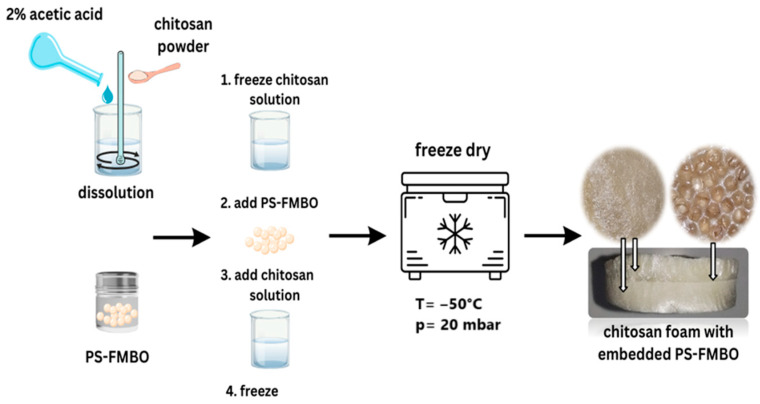
Procedure for the preparation of chitosan foams.

A turbo mixer (VELP Scientifica, OV5 Homogenizer, Milano, Italy) was used to enable fast dissolution of the chitosan. After complete dissolution of the chitosan, one part of the solution (10 mL) was poured into a glass beaker and left to freeze at ~−50 °C for 2 h. The preparation process and formed foams are presented in Figure 2.

Three grams of the PS-FMBO beads were placed on top of the frozen layer of chitosan, and a further 10 mL of chitosan solution was added to the glass beaker. The beads were thus completely immersed in the chitosan solution. This process was followed by an additional freezing step under the same conditions as the previous one. Once freezing of all layers was completed, the glass beaker was placed in the freeze dryer at −50 °C and 20 mbar overnight for the complete elimination of liquids. Finally, the chitosan foams with embedded PS-FMBO beads were obtained for use in the sound absorption tests.

### 2.5. Sound Absorption Test

**Figure 3:**
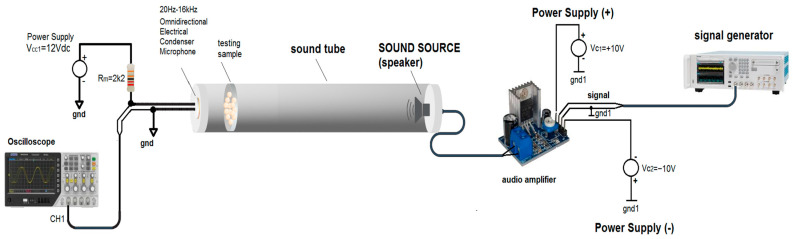
Experimental setup for sound absorption tests.

Due to the lack of direct measurement of sound absorption by the impedance tube method, an alternative approach was developed in the laboratory, in which the sound transmission loss was analyzed. This configuration measures how much sound passes through the material, rather than being absorbed or reflected. Figure 3 shows the setup used in the experiments.

The experimental setup consisted of a long cylindrical plastic tube, with a loudspeaker (1 W, 8 Ω) at one end to generate sound waves and a microphone (20 Hz–16 kHz/120 dB) at the other end to receive the transmitted sound waves. The tested foam was placed close to the second end of the tube, where the microphone was mounted, and it completely covered the cross-section of the tube.

### 2.6. Methods

#### 2.6.1. Characterization Techniques

The chemical characterization of PS-FMBO before and after stabilization with quicklime and cement was performed using Fourier transform infrared spectroscopy (FTIR) on a Nicolet iS20 FTIR spectrometer (Thermo Fisher Scientific, Waltham, MA, USA).

#### 2.6.2. Leaching Tests

Leaching tests were conducted on the solidified and stabilized (S/S) samples at different aging intervals: 7 days, 28 days, and 3 months following the S/S treatment. These specific time points were selected to assess the influence of pozzolanic reactions, which occur within the first 7 to 28 days and contribute to the reduction of metal mobility and the enhancement of mechanical stability. Additionally, the long-term stability of immobilized metals was evaluated after 3 months to investigate potential leaching behaviour over extended periods, a factor that remains largely unexplored in the literature.

For the German standard leaching test (DIN 3841-4 S^4^), the samples were ground to a maximum particle size of 10 mm, as specified in the relevant standard. Leaching was performed using deionized water at a liquid-to-solid (L/S) ratio of 10:1 (L/kg) over a duration of 24 h under continuous agitation. The resulting suspensions were subsequently filtered using 0.45 µm membrane filters, and the pH of the filtrates was recorded. The concentrations of target metals in the leachates were quantified using ICP-MS. Each leaching test analysis was conducted in triplicate, and mean values were reported with standard deviations (SDs).

The toxicity characteristic leaching procedure (TCLP) was performed following the United States Environmental Protection Agency (USEPA) method 1311 [41]. This test involves the extraction of metals from the S/S matrices by mixing 100 g of the sample with the extraction solution. Extraction fluid 1 was selected, as the initial pH of all samples was ≤5. The leaching procedure was carried out at an L/S ratio of 20:1 for 18 h under continuous agitation. After extraction, the pH of the resulting leachates was measured, and the solutions were filtered through 0.45 µm membrane filters. The concentrations of metals in the filtrates were determined by using the ICP-MS technique (Agilent Technologies 7700× Series ICP-MS, Tokyo, Japan).

#### 2.6.3. Analytical Methods

pH measurements were performed using an InoLab pH/ION 735 instrument (WTW GmbH, Weilheim, Germany). The concentrations of arsenic, iron, and manganese in the samples were quantified using inductively coupled plasma mass spectrometry (ICP-MS). The method detection limits for arsenic, iron, and manganese were 0.001 mg/L.

## 3. Results

### 3.1. Regeneration and Reuse of PS-FMBO

The PS-FMBO regeneration performance was first studied in batch operation mode. Lab-scale tests were conducted in order to evaluate the desorption of As(III) and As(V) from PS-FMBO using different eluents at different concentrations. Regeneration methods were investigated over three consecutive adsorption–desorption cycles. The desorption of As(III) and As(V) from PS-FMBO was performed by applying different concentrations of NaOH as well as the mixture of NaOH, NaCl, and NaOCl [42].

**Figure 4:**
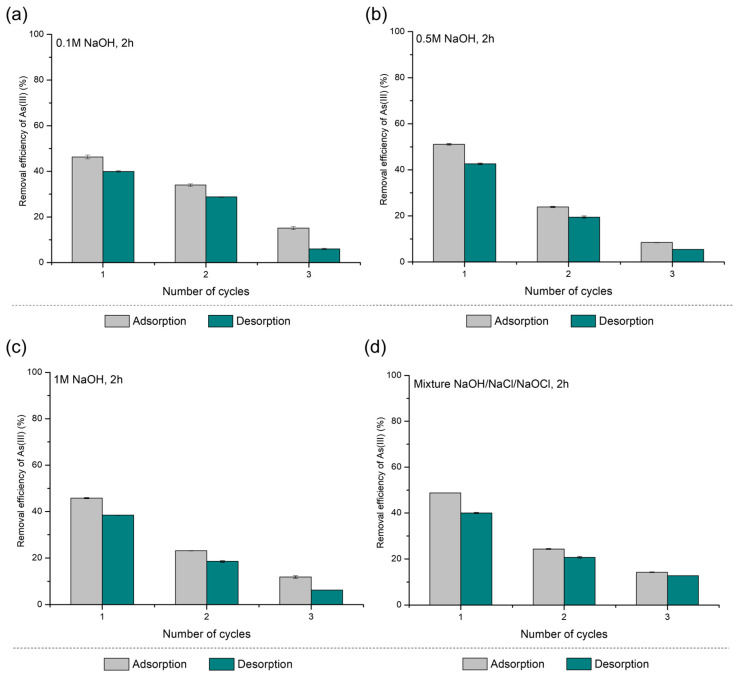
Results of As(III) adsorption/desorption cycles on PS-FMBO.

The results for As(III) adsorption/desorption cycles on PS-FMBO are presented in Figure 4. PS-FMBO exhibited a decline in arsenic removal efficiency with increasing cycle number. After the first adsorption/desorption cycle using 0.1 M NaOH, As(III) adsorption efficiency decreased by 12%. Increasing the NaOH concentration (0.5 M and 1 M) further reduced the adsorption efficiency, with removal efficiency decreasing by 30% and 20%, respectively, after the first desorption cycle. Similarly, using a mixture of NaOH, NaCl, and NaOCl for desorption resulted in a 23% decrease in removal efficiency after the first cycle.

These findings suggest that stronger desorption agents may contribute to structural or chemical modifications of PS-FMBO, potentially reducing its adsorption capacity in subsequent cycles. The results presented in Figure 4 indicate that among the tested eluents, 0.1 M NaOH provides the best regeneration performance, maintaining higher adsorption efficiency over repeated cycles. Further investigation into optimizing regeneration conditions is necessary to enhance the long-term reusability of PS-FMBO for arsenic removal applications.

**Figure 5:**
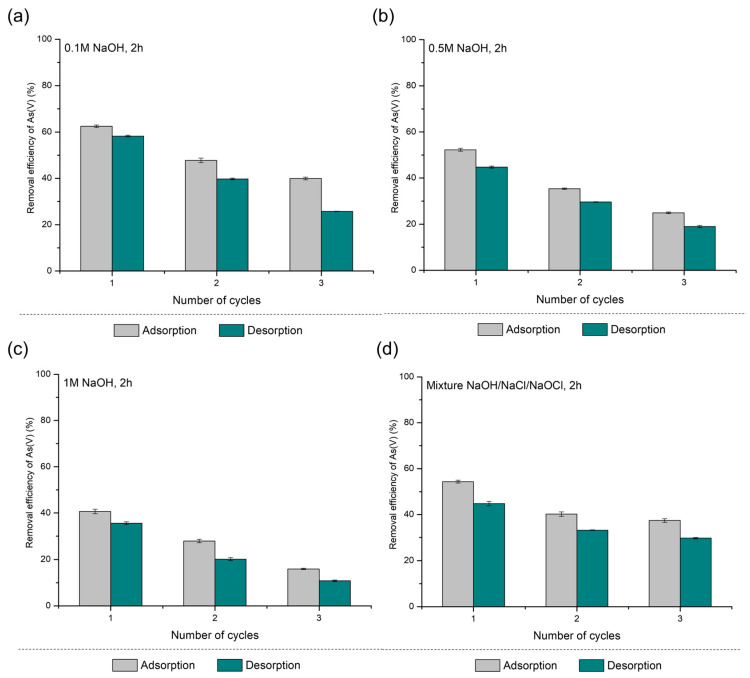
Results of As(V) adsorption/desorption cycles on PS-FMBO.

The regeneration performance of PS-FMBO for As(V) removal was also evaluated, and the results are presented in Figure 5. The data again indicates a progressive decline in adsorption efficiency with an increasing number of regeneration cycles. After the third cycle, As(V) adsorption efficiency on PS-FMBO decreased by 20% with 0.1 M NaOH and by 25% for both 0.5 M and 1 M NaOH. Consequently, the removal efficiency of As(V) after three cycles was reduced to 40%, 25%, 17%, and 38% for 0.1 M NaOH, 0.5 M NaOH, 1 M NaOH, and the NaOH–NaCl–NaOCl mixture, respectively. This represents a significant decline compared to the initial adsorption efficiencies of 60%, 50%, 40%, and 55% observed after the first adsorption cycle.

An increase in NaOH concentration led to a substantial reduction in As(V) adsorption on PS-FMBO, indicating that highly alkaline conditions negatively impact the adsorbent’s structural and functional integrity. The decline in As(V) adsorption efficiency on PS-FMBO can be attributed to the excessive deprotonation of surface functional groups, the disruption of adsorption-active sites, and the potential leaching of metal oxides, which together undermine the material’s structural integrity and regeneration potential. In contrast, the NaOH–NaCl–NaOCl mixture maintained a similar adsorption efficiency to 0.1 M NaOH, suggesting that aggressive alkaline conditions are not necessary for effective regeneration. These findings highlight 0.1 M NaOH as the most suitable regenerant, offering optimal desorption performance while minimizing adsorbent degradation, thereby extending the operational lifespan of PS-FMBO. The findings are consistent with our research focused on the adsorption/desorption performance of PE-FMBO and PET-FMBO nanocomposites, as presented in Nikić et al. (2024) [12]. In that study, Nikić et al. (2024) found that the application of 0.1 M NaOH was the most effective strategy for regenerating polymer-based nanocomposites after As(III) and As(V) adsorption [12].

The successive regeneration cycles led to a gradual reduction in the adsorption capacity of PS-FMBO, which can be attributed to both physical and chemical factors. A primary factor influencing this behaviour is the gradual saturation or deactivation of the material’s surface adsorption sites. As adsorbed species accumulate and surface fouling occurs during the arsenic adsorption and desorption cycles, the availability of active sites diminishes [43]. Furthermore, mechanical stress and chemical alterations that occur during these cycles can weaken the nanocomposites, resulting in a decrease in their overall adsorption capacity.

The reduction in As(V) adsorption at higher pH values is associated with the shift in predominant arsenic species to HAsO_4_^2−^, which possesses a higher negative charge. Furthermore, extended exposure to highly alkaline environments, such as NaOH solutions, can induce significant alterations in the surface chemistry of the adsorbent. Changes in surface charge and functional groups, crucial for arsenic binding, result in a more negatively charged surface, enhancing the electrostatic repulsion between As(V) species and the adsorptive sites [44]. Moreover, physical degradation, including breakdown of the porous structure or leaching of metal components, can further reduce the adsorbent’s efficiency [42,45,46,47]. These factors collectively contribute to a significant loss of arsenic removal capacity, highlighting the need for optimized regeneration strategies to maintain long-term adsorbent effectiveness [43,48].

### 3.2. Stabilization of PS-FMBO Nanocomposites

#### 3.2.1. Physicochemical Characteristics of S/S PS-FMBO Nanocomposite

**Figure 6:**
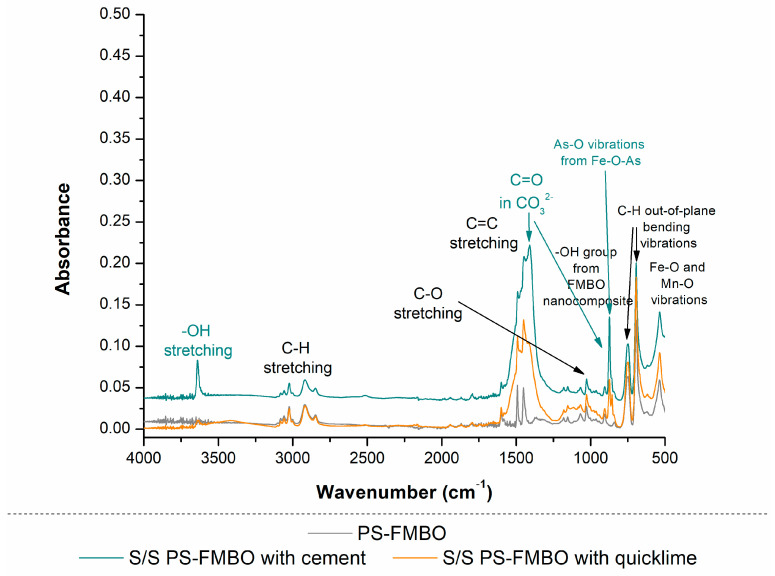
FTIR spectrum of PS-FMBO before and after stabilization with cement and quicklime.

The FTIR spectra of PS-FMBO (Figure 6) exhibited distinct absorption peaks at 3059, 3024, and 2920 cm^−1^, corresponding to aromatic C-H stretching vibrations. Additionally, peaks at 1601, 1491, and 1451 cm^−1^ were assigned to aromatic C=C stretching, confirming the presence of benzene rings. The absorption bands at 755 and 697 cm^−1^, associated with C-H out-of-plane bending vibrations, indicate mono-substituted benzene structures [49,50].

Following the modification of PS with Fe–Mn binary oxides, new absorption peaks appeared in the 419–775 cm^−1^ range, attributed to Fe-O and Mn-O vibrations, indicating the successful incorporation of metal oxides. Moreover, additional peaks in the 965–1244 cm^−1^ range were observed, corresponding to the vibrations of surface-bound -OH groups on FMBO nanocomposites. These spectral changes confirm the successful surface functionalization of PS with FMBO, demonstrating the formation of a stable polymer–metal oxide composite.

The PS-FMBO nanocomposites stabilised with cement and quicklime were also analysed by FTIR, as presented in Figure 6. In the FTIR spectra of the PS-FMBO stabilized with cement and quicklime, besides specific vibrations for PS-FMBO, vibrations for functional groups characteristic of cement and quicklime were detected. Vibrations detected at 520 cm^−1^, 657 cm^−1^_,_ and 923 cm^−1^ are specific bands for the tetrahedral SiO_4_ group in cement. Additionally, the presence of the trigonal planar carbonate group CO_3_ was also detected, based on the C=O bond with bands at 875 and 1411–1473 cm^−1^ and C–H group at 2895 cm^−1^ [51]. The FTIR spectrum of PS-FMBO stabilized with quicklime revealed a distinct absorption band at 871 cm^−1^, corresponding to the out-of-plane vibrational modes of carbonate (CO_3_^2−^) groups. Strong C–O stretching vibrations were observed at 1064 cm^−1^, and the asymmetric stretching of the C=O bond in CO_3_^2−^ ions was evident at 1451 cm^−1^, confirming the presence of carbonate species [52]. A sharp peak at 3633 cm^−1^ was attributed to hydroxyl (-OH) stretching, indicating the presence of hydroxide species, likely due to surface-bound water molecules. In addition, the FTIR analysis of the arsenic-saturated PS-FMBO stabilized with cement and lime revealed distinctive As-related bands observed at 830 cm^−1^, corresponding to valence vibrations of As-O (from Fe-O-As) [51,53]. This indicates that after arsenic adsorption, the adsorbent was successfully stabilized using these two immobilization agents.

#### 3.2.2. Leaching Characteristics and Environmental Risk Assessment of S/S PS-FMBO Nanocomposite

**Figure 7:**
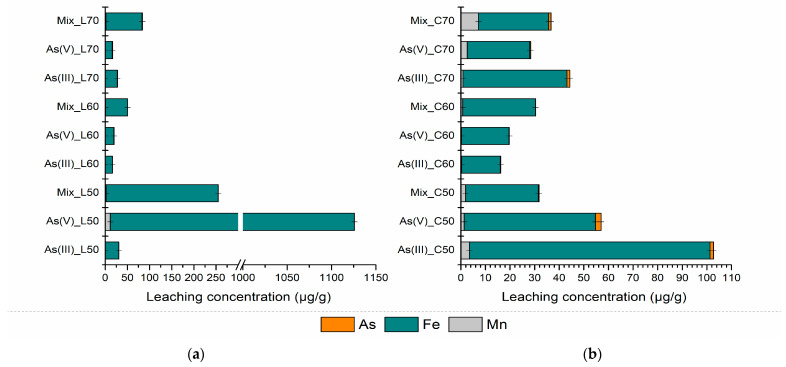
Results of As, Fe, and Mn contents of leachates after performing the DIN test on PS-FMBO mixtures with (**a**) quicklime and (**b**) cement after 3 months of S/S treatment.

The PS-FMBO nanocomposite investigated in this study was saturated with As(III), As(V), and a mixture of As(III) and As(V). The potential environmental and toxicological impact of these contaminants was systematically assessed through leaching experiments. The results of the DIN test (Figure 7a and Table 2) were evaluated in accordance with national regulations for waste classification as inert, non-hazardous, or hazardous [54] and compared with the threshold values established by the European Union [55].

PS-FMBO, when combined with cement in ratios of 50:50, 40:60, and 30:70, was classified as inert waste concerning As, Fe, and Mn, based on both European Union directives and Serbian legislation [54]. The DIN test results (Figure 7a and Table 2) indicate that Fe exhibited the highest leaching levels, regardless of the mixture ratio used in building material composition. Moreover, the leaching behaviour of Fe implied a decreasing trend over time, with concentrations declining from 1125 µg/g to 369 µg/g after a three-month aging period. This reduction in Fe leachability supports the classification of the material as non-hazardous waste.

Additionally, the leaching concentration of manganese also decreases progressively over time, with lower values observed after 7 days, 28 days, and 3 months, indicating a stabilization process that limits Mn release. The results of the DIN leaching test further demonstrate that arsenic exhibits the lowest leaching concentration among the analyzed elements. Even after 3 months (Figure 7a) of stabilization, the leached arsenic concentration remains below the established emission limit values, confirming the material’s high efficiency in immobilizing arsenic [54]. The findings indicate that the stabilization process effectively captures both As(III) and As(V) with equal efficiency, regardless of whether they are present individually or as a mixture. This highlights the robustness of the material in binding arsenic in different oxidation states, reducing the risk of its release under environmental conditions. The long-term stability of arsenic and manganese further underscores the potential of this material for safe and sustainable waste management applications.

The lowest leaching concentrations of As, Fe, and Mn were observed when the PS-FMBO to immobilization agent ratio is 30:70, suggesting that a higher proportion of the immobilization agent enhances the stabilization of these elements. However, even when the ratio is adjusted to 50 wt%, no significant increase in the leaching concentrations of the elements of interest is observed. This indicates that the stabilization efficiency of the material remains consistent across different ratios, ensuring reliable containment of potentially hazardous elements. The fact that no major variations in leaching behaviour occur between the 30:70, 40:60, and 50:50 ratios suggests that 50 wt% may represent an optimal composition. This balance provides sufficient immobilization while potentially improving the mechanical and structural properties of the material, making it more suitable for practical applications. The stability of this mixture under various conditions underscores its feasibility for use in construction-related purposes, such as in the production of bricks, concrete additives, or other building materials where long-term durability and environmental safety are essential. Furthermore, the observed stabilization across different mixture ratios suggests that the immobilization process is robust and effective in maintaining the integrity of the material. The minimal variation in leaching behaviour implies that the chemical binding mechanisms responsible for immobilization, such as adsorption, precipitation, or encapsulation, remain effective even with variations in composition. This is particularly important for industrial applications, where material formulations may need to be adjusted based on specific requirements without compromising safety and environmental standards [21,56].

Significantly lower leaching concentrations of As, Fe, and Mn were observed in the results of the DIN test following the application of cement as the immobilization agent (Figure 7b and Table 2). This suggests that cement effectively reduces the mobility of these potentially toxic elements, thereby minimizing their potential environmental impact. Similar to stabilization using quicklime, the leaching tests indicate that after a stabilization period of 3 months, Fe exhibits the highest leaching concentration, while As and Mn show lower levels. This indicates that, despite cement’s efficacy in immobilizing the majority of contaminants, iron may exhibit comparatively higher mobility due to its specific chemical properties or the particular phases it forms within the matrix [26,27,57].

A clear trend was observed whereby increasing the quantity of cement in the matrix results in a reduction in the leaching, highlighting the dose-dependent nature of cement’s immobilizing effect. The observed reduction in leaching concentrations can be attributed to the increased availability of surface sites for chemical binding between the cement and contaminants, as well as the formation of stable mineral phases that encapsulate the contaminants. Higher cement contents thus enhance the material’s capacity to stabilize the contaminants, leading to reduced leachability.

Importantly, even at a 50:50 PS-FMBO to cement ratio, the leaching concentrations of As, Fe, and Mn remain below the regulatory emission limit values for environmental discharge [54]. This indicates that this particular composition provides a sufficient level of stabilization to meet environmental safety standards. Therefore, PS-FMBO stabilized with cement at a 50:50 ratio can be safely considered for applications such as the construction of containment barriers at landfills, waste processing facilities, or other infrastructure where long-term stabilization and containment of contaminants is essential [58,59]. The stability of the material at this ratio ensures that it will not pose a significant environmental risk while also offering potential for safe, sustainable use in the construction industry. The findings support the viability of using cement as an effective immobilization agent for the stabilization of heavy metals, particularly As, Fe, and Mn, in waste management and construction applications. The reduction in leaching potential, coupled with the ability to achieve compliance with environmental regulations, demonstrates the potential for PS-FMBO to be utilized in various applications, contributing to the development of sustainable, environmentally responsible solutions for waste stabilization and containment [54,59].

**Figure 8:**
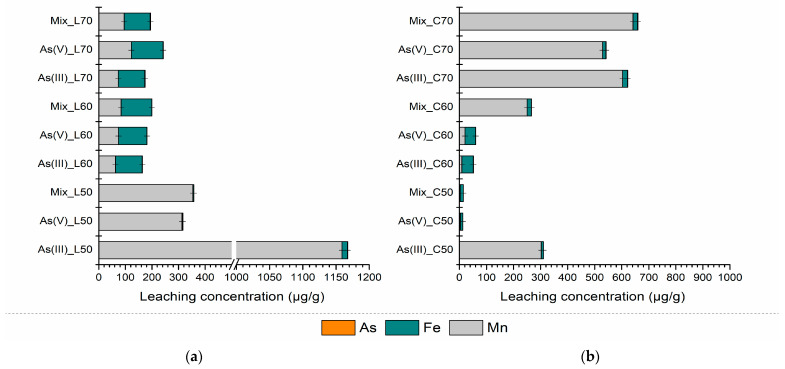
TCLP test results of PS-FMBO mixtures with (**a**) quicklime and (**b**) cement after 3 months of S/S treatment.

The toxicity characteristic leaching procedure (TCLP) is an analytical method employed to assess the mobility of inorganic and organic analytes in solid, liquid, and multiphase waste materials. This test simulates landfill conditions and, based on the concentrations of the tested components in the leachate, determines whether the waste poses a risk to the environment. Figure 8 and Table 3 present the results of the TCLP test conducted on mixtures of quicklime and concrete over three aging periods (7 days, 28 days, and 3 months of aging).

For both mixtures, a linear decrease in the leached concentrations of Fe, Mn, and As was observed as aging progressed. Importantly, these concentrations remained below the regulatory limit values established by the Rulebook on categories, testing, and classification of waste [54]. The results presented in Figure 8 indicate that the mixtures do not exhibit toxic characteristics, as their leaching behaviour complies with environmental safety standards. Therefore, it can be concluded that these mixtures do not pose an environmental hazard in terms of leaching.

These findings substantiate the effectiveness of the stabilization and solidification treatment, even 3 months after the stabilization/solidification (S/S) application. Monitoring the leached concentrations of Fe, Mn, and As under more aggressive leaching conditions (compared to the DIN test) confirmed that the values remained significantly below the emission limit values. In a related study, Aiken et al. (2020) demonstrated that fly ash-based geopolymers exhibited superior resistance to acetic and lactic acids, as evidenced by smaller mass losses [60]. This enhanced resistance was attributed to the higher stability of the geopolymer gel in organic acids, which is further supported by the increased porosity of the material, allowing for more efficient inward diffusion of acids without significantly compromising the structural integrity.

Overall, the findings highlight the potential of PS-FMBO-based materials as a sustainable and environmentally responsible alternative for waste stabilization and construction applications. By providing both effective contaminant immobilization and structural stability, these materials could contribute to the development of eco-friendly construction solutions, reducing the environmental impact of industrial waste while promoting the principles of the circular economy.

### 3.3. Stabilization of Chitosan Foam

#### 3.3.1. Physicochemical Characteristics of Chitosan Foam

**Figure 9:**
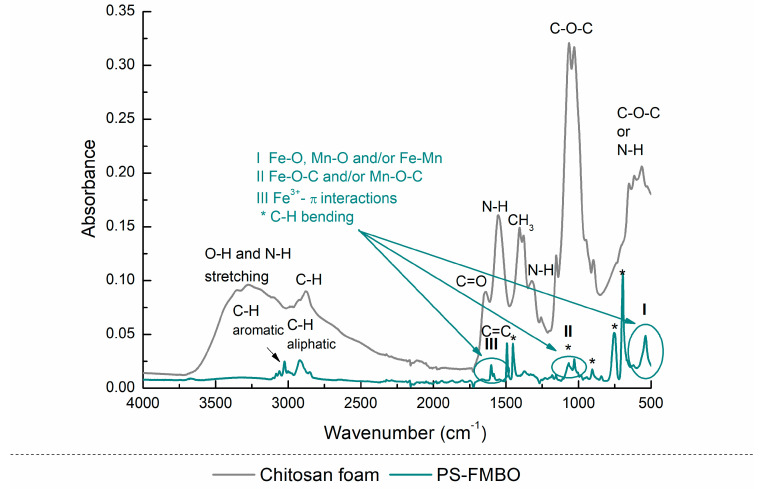
FTIR analysis of chitosan foams and PS-FMBO beads.

FTIR spectra of the chitosan foam and PS-FMBO beads are presented in Figure 9. According to the literature data, all characteristic bands of chitosan are identified and marked on the diagram. The spectrum obtained for PS beads shows the characteristic bonds found in polystyrene and the bonds formed between FMBO particles and polystyrene.

The strong absorption band at 540 cm^−1^ can thus be assigned to the Fe–O, Mn–O, and/or Fe–Mn stretching mode; Fe–O–C or Mn–O–C stretching modes can be observed in the region 1000–1150 cm^−1^ to, and the band at 1600 cm^−1^ can be attributed to the π–π interactions in the benzene ring, confirming the presence of FMBO particles on the polystyrene beads [50,51,53].

#### 3.3.2. Sound Absorption Test

**Figure 10:**
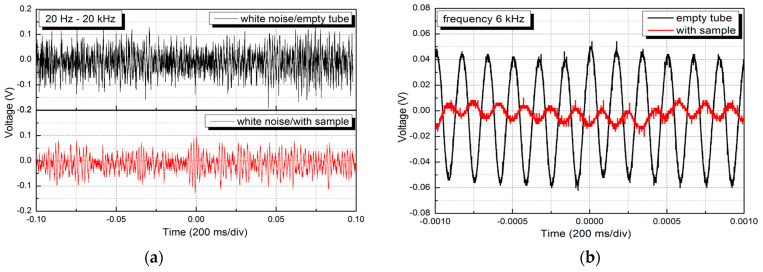
Sound absorption test results of chitosan films under the influence of white noise (**a**), absorption test results at 6 kHz (**b**).

The first sound absorption test was performed using white noise, which contains a wide range of frequencies, from 20 Hz to 20 kHz. Figure 10a shows the signal obtained from the oscilloscope, (1) when the testing tube is empty, and (2) when the sample is placed in the tube. The change in intensity of the two signals obtained by the microphone is readily apparent. Fast Fourier transform has shown that the highest absorption potential of the tested material is in the range of frequencies from 600 Hz to 7 kHz. According to the literature, most porous materials like the one investigated in this work are effective in this range [61,62,63]. These frequencies include sounds obtained from speech and indoor noise (250 Hz–3 kHz) as well as sounds from musical instruments, communication devices, and ultrasonic devices (2–10 kHz).

The 6 kHz frequency is significant in audiology as it can be used as a marker for hearing impairment due to loud noise exposure. This frequency was therefore selected for further analysis of sound absorption. The spectra of 6 kHz sound waves emitted from the loudspeaker were recorded again, with the sample placed in the tube and with the tube empty (Figure 10b). The absorption coefficient was calculated using the equation, α = 1 − (It/Ii), where It and Ii are the intensities of the transmitted and incident sound waves, respectively. The intensities of the sound waves are calculated from the equation: I = P2/ρ*c, where P is the sound pressure in Pa, ρ is the density of the air, approximately 1.21 kg/m^3^, and c is the speed of sound in the air, approximately 343 m/s. According to the specification of the microphone used in the experiment, the sound pressure was calculated by P = V/S, where V is the measured voltage in V, and S is the sensitivity of the microphone, 5 mV/Pa. The absorption coefficient thus obtained is near full absorption with a value of ~0.99. The explanation for these results can be found in the highly porous structure of the chitosan foams that enables efficient absorption of the sound waves trapped in its structure. On the other hand, PS-FMBO embedded beads additionally scatter sound waves and improve sound absorption of this composite material [61,63]. However, a more detailed analysis should be carried out at various frequencies and using an impedance tube method to get the whole absorption potential of this material.

#### 3.3.3. Chemical Stability of Chitosan Foams

**Figure 11:**
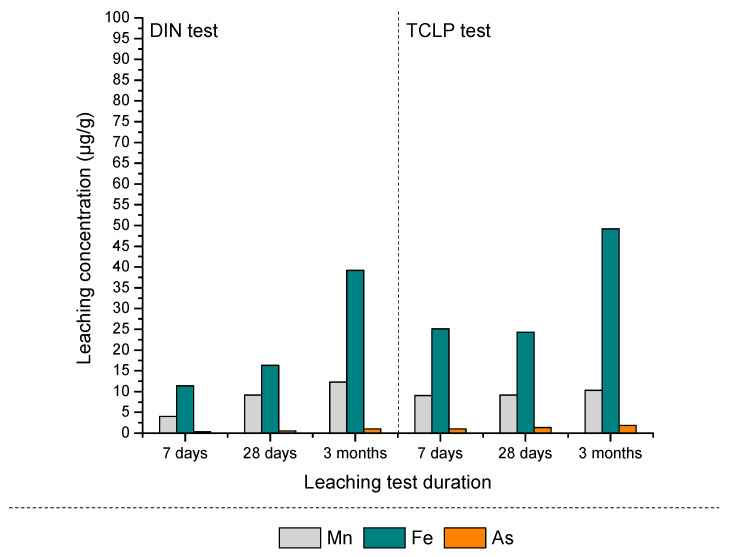
DIN and TCLP test results of chitosan foam after 7 days, 28 days, and 3 months.

The chemical stability of chitosan-based foams was systematically assessed through the application of standard leaching tests conducted at three different time intervals: 7 days, 28 days, and 3 months. As with the PS-FMBO material, two distinct leaching tests were employed for this purpose: the DIN test and the toxicity characteristic leaching procedure (TCLP) test [41]. These tests were chosen to evaluate both the short-term and long-term leaching behaviour of the foam, considering both mild and more aggressive leaching conditions. The results from these tests are presented in Figure 11.

Upon analyzing the data shown in Figure 11, it is evident that the leaching concentrations of arsenic (As), iron (Fe), and manganese (Mn)—the key components of the chitosan-based foam—increase slightly as the foam ages. This increase, however, remains minimal and within acceptable ranges. This suggests that while there may be some gradual release of these elements over time, the material continues to exhibit stability under typical environmental conditions [64,65]. Furthermore, the use of the TCLP test, which is a more aggressive and invasive method designed to simulate potential extreme environmental conditions, provided critical insight into the long-term environmental behaviour of the material. The results of the TCLP test indicate that, despite the more severe conditions it simulates, the leaching concentrations of As, Fe, and Mn remained within the regulatory emission limit values. This finding is particularly significant because the TCLP test is intended to replicate the behaviour of waste materials when exposed to potentially hazardous leaching environments, such as those found in landfills or contaminated sites [60,66,67].

The fact that the leaching concentrations of the tested elements remained below the established limit values further highlights the material’s safety and its suitability for real-world applications [54]. Specifically, these results suggest that chitosan-based foam can be safely used in everyday life, particularly as an acoustic insulating material. This application is important because such materials are often used in environments where long-term exposure to airborne particles or contaminants might be a concern. The low leaching potential demonstrated by the foam indicates that it will not contribute significantly to environmental pollution or pose health risks over extended periods of use.

In conclusion, both the DIN and TCLP leaching tests demonstrated that the chitosan-based foam exhibits satisfactory chemical stability and minimal leaching of As, Fe, and Mn, even under aging conditions. The slight increase in leaching observed over time remains well within the regulatory emission limits. The results affirm that the material does not pose an environmental or health risk, even under more aggressive leaching conditions simulated by the TCLP test. Therefore, the chitosan-based foam can be safely utilized as an acoustic insulating material in practical applications, offering a sustainable and environmentally compliant solution for everyday use [68,69].

## 4. Conclusions

This study systematically evaluated the regeneration and reusability of PS-FMBO for As removal through multiple batch adsorption–desorption cycles. The results demonstrated a gradual decline in arsenic removal efficiency with successive regeneration cycles, which was attributed to the degradation of the adsorbent’s structural and chemical integrity. Among the eluents tested, 0.1 M NaOH exhibited the most effective regeneration performance, preserving the highest arsenic adsorption capacity across multiple cycles. Conversely, stronger desorption agents, such as higher concentrations of NaOH and NaOH-NaCl-NaOCl mixtures, led to a more pronounced decrease in arsenic removal efficiency, suggesting that optimal regeneration conditions are crucial to minimizing adsorbent degradation and enhancing long-term reusability for arsenic remediation.

In addition, the stabilization of used PS-FMBO nanocomposites through the incorporation of cement and quicklime was investigated. The DIN and TCLP leaching tests revealed that the stabilized composites successfully immobilized arsenic (As(III) and As(V)), iron (Fe), and manganese (Mn) with minimal leaching, indicating their compliance with environmental safety standards. These findings suggest that PS-FMBO stabilized with cement or quicklime can be used in waste stabilization, and also demonstrated that such stabilized material can find its application in construction applications, providing a safe and effective solution for the immobilization of hazardous contaminants. For chitosan-based foams, the results of leaching tests demonstrated excellent chemical stability, with negligible increases in the leaching of As, Fe, and Mn over time, all of which remained within regulatory limits. These results confirmed that the chitosan-based foams maintained their structural integrity and chemical stability even under aggressive leaching conditions, making them suitable for use in various industrial applications, particularly as environmentally friendly acoustic insulating materials. In summary, the findings presented in this study highlight the potential of PS-FMBO and chitosan-based foams as sustainable and effective materials for environmental applications. PS-FMBO offers a promising option for arsenic removal with optimized regeneration protocols, while both PS-FMBO stabilized composites and chitosan foams exhibit excellent stability and minimal environmental impact. These materials show significant promise for use in waste management, construction, and other industrial applications, offering a viable solution to address environmental concerns related to hazardous contaminant removal and immobilization.

## Figures and Tables

**Table 1:** Composition of PS-FMBO mixtures with quicklime and cement.

Samples	Sample Name	Mass (g)		
		Quicklime	Cement	PS-FMBO
As(III)_50:50-PS-FMBO:L	As(III)_L50	10	-	10
As(V)_50:50-PS-FMBO:L	As(V)_L50	10	-	10
As(III) + As(V)_50:50-PS-FMBO:L	Mix_L50	10	-	10
As(III)_40:60-PS-FMBO:L	As(III)_L60	12	-	8
As(V)_40:60-PS-FMBO:L	As(V)_L60	12	-	8
As(III) + As(V)_40:60-PS-FMBO:L	Mix_L60	12	-	8
As(III)_30:70-PS-FMBO:L	As(III)_L70	14	-	6
As(V)_30:70-PS-FMBO:L	As(V)_L70	14	-	6
As(III) + As(V)_30:70-PS-FMBO:L	Mix_L70	14	-	6
As(III)_50:50-PS-FMBO:C	As(III)_C50	-	10	10
As(V)_50:50-PS-FMBO:C	As(V)_C50	-	10	10
As(III) + As(V)_50:50-PS-FMBO:C	Mix_C50	-	10	10
As(III)_40:60-PS-FMBO:C	As(III)_C60	-	12	8
As(V)_40:60-PS-FMBO:C	As(V)_C60	-	12	8
As(III) + As(V)_40:60-PS-FMBO:C	Mix_C60	-	12	8
As(III)_30:70-PS-FMBO:C	As(III)_C70	-	14	6
As(V)_30:70-PS-FMBO:C	As(V)_C70	-	14	6
As(III) + As(V)_30:70-PS-FMBO:C	Mix_C70	-	14	6

**Table 2:** Leaching of As, Fe, and Mn from the S/S PS-FMBO nanocomposite mixtures with immobilization agents after 7 and 28 days, according to the DIN test.

Immobilization Agents	Quicklime (L)				Cement (C)		
Samples	Leaching Concentration Metals in µg/g			Samples	Leaching Concentration Metals in µg/g		
	As	Fe	Mn		As	Fe	Mn
As(III)_L50 7 d	1.21 ± 0.016	25.8 ± 0.018	1.11 ± 0.001	As(III)_C50 7 d	0.47 ± 0.008	24.3 ± 0.010	0.58 ± 0.016
As(III)_L50 28 d	0.28 ± 0.010	29.5 ± 0.010	0.38 ± 0.009	As(III)_C50 28 d	3.26 ± 0.006	49.8 ± 0.012	4.10 ± 0.011
As(V)_L50 7 d	4.78 ± 0.009	364.1 ± 0.005	4.05 ± 0.015	As(V)_C50 7 d	3.38 ± 0.015	97.9 ± 0.005	84.6 ± 0.010
As(V)_L50 28 d	3.71 ± 0.010	758.4 ± 0.011	12.2 ± 0.009	As(V)_C50 28 d	1.33 ± 0.020	84.7 ± 0.019	1.37 ± 0.008
Mix_L50 7 d	2.78 ± 0.011	109.4 ± 0.018	10.9 ± 0.013	Mix_C50 7 d	0.55 ± 0.004	20.8 ± 0.011	0.47 ± 0.009
Mix_L50 28 d	1.42 ± 0.019	227.1 ± 0.019	2.16 ± 0.017	Mix_C50 28 d	0.61 ± 0.002	30.2 ± 0.005	1.05 ± 0.015
As(III)_L60 7 d	1.42 ± 0.019	53.9 ± 0.014	0.70 ± 0.012	As(III)_C60 7 d	0.74 ± 0.016	13.0 ± 0.018	38.5 ± 0.010
As(III)_L60 28 d	0.20 ± 0.007	15.8 ± 0.020	0.39 ± 0.018	As(III)_C60 28 d	2.34 ± 0.020	49.0 ± 0.014	5.29 ± 0.004
As(V)_L60 7 d	2.82 ± 0.007	67.3 ± 0.008	0.52 ± 0.009	As(V)_C60 7 d	2.06 ± 0.016	107.4 ± 0.019	0.60 ± 0.011
As(V)_L60 28 d	0.13 ± 0.011	19.3 ± 0.016	0.33 ± 0.006	As(V)_C60 28 d	0.68 ± 0.006	28.4 ± 0.009	0.49 ± 0.016
Mix_L60 7 d	0.25 ± 0.008	14.8 ± 0.003	2.00 ± 0.007	Mix_C60 7 d	0.15 ± 0.013	14.8 ± 0.016	1.38 ± 0.019
Mix_L60 28 d	0.09 ± 0.015	49.6 ± 0.008	0.82 ± 0.003	Mix_C60 28 d	0.55 ± 0.009	28.0 ± 0.001	1.52 ± 0.016
As(III)_L70 7 d	1.13 ± 0.004	23.9 ± 0.006	0.64 ± 0.013	As(III)_C70 7 d	2.68 ± 0.018	27.7 ± 0.003	0.63 ± 0.019
As(III)_L70 28 d	0.44 ± 0.016	22.4 ± 0.012	0.20 ± 0.013	As(III)_C70 28 d	1.31 ± 0.002	34.7 ± 0.009	1.01 ± 0.003
As(V)_L70 7 d	2.30 ± 0.016	56.4 ± 0.019	0.69 ± 0.018	As(V)_C70 7 d	3.43 ± 0.002	28.3 ± 0.008	7.22 ± 0.007
As(V)_L70 28 d	0.81 ± 0.003	11.7 ± 0.005	0.44 ± 0.002	As(V)_C70 28 d	0.44 ± 0.011	42.7 ± 0.015	1.49 ± 0.018
Mix_L70 7 d	1.91 ± 0.018	45.6 ± 0.014	0.58 ± 0.002	Mix_C70 7 d	1.61 ± 0.002	20.2 ± 0.006	0.32 ± 0.007
Mix_L70 28 d	1.38 ± 0.002	62.6 ± 0.009	1.53 ± 0.006	Mix_C70 28 d	3.48 ± 0.008	18.5 ± 0.009	8.43 ± 0.002

**Table 3:** Leaching of As, Fe, and Mn from the S/S PS-FMBO nanocomposite mixtures with quicklime (L) and cement (C) according to the TCLP test after 7 and 28 days.

Immobilization Agents	Quicklime (L)				Cement (C)		
Samples	Leaching Concentration Metals in µg/g			Samples	Leaching Concentration Metals in µg/g		
	As	Fe	Mn		As	Fe	Mn
As(III)_L50 7 d	0.48 ± 0.010	9.56 ± 0.008	1551.1 ± 0.019	As(III)_C50 7 d	0.35 ± 0.018	11.4 ± 0.016	4.01 ± 0.001
As(III)_L50 28 d	0.34 ± 0.007	9.01 ± 0.005	1231.7 ± 0.003	As(III)_C50 28 d	0.53 ± 0.016	10.8 ± 0.011	4.16 ± 0.006
As(V)_L50 7 d	4.47 ± 0.009	5.94 ± 0.006	423.0 ± 0.007	As(V)_C50 7 d	0.39 ± 0.010	11.2 ± 0.010	5.97 ± 0.009
As(V)_L50 28 d	0.15 ± 0.011	5.27 ± 0.011	326.9 ± 0.018	As(V)_C50 28 d	0.30 ± 0.003	10.0 ± 0.008	4.00 ± 0.007
Mix_L50 7 d	1.23 ± 0.005	5.70 ± 0.012	510.7 ± 0.007	Mix_C50 7 d	0.21 ± 0.005	12.7 ± 0.009	5.64 ± 0.015
Mix_L50 28 d	0.24 ± 0.008	5.12 ± 0.018	498.4 ± 0.002	Mix_C50 28 d	0.32 ± 0.008	12.0 ± 0.015	3.10 ± 0.003
As(III)_L60 7 d	2.86 ± 0.014	114.0 ± 0.019	94.6 ± 0.011	As(III)_C60 7 d	0.46 ± 0.011	47.9 ± 0.010	9.72 ± 0.009
As(III)_L60 28 d	0.51 ± 0.015	102.3 ± 0.005	80.75 ± 0.018	As(III)_C60 28 d	0.30 ± 0.006	42.6 ± 0.004	9.15 ± 0.013
As(V)_L60 7 d	1.29 ± 0.009	122.7 ± 0.005	106.5 ± 0.008	As(V)_C60 7 d	1.47 ± 0.018	43.6 ± 0.011	15.4 ± 0.013
As(V)_L60 28 d	0.66 ± 0.004	118.5 ± 0.014	94.1 ± 0.010	As(V)_C60 28 d	0.75 ± 0.012	39.6 ± 0.016	20.3 ± 0.013
Mix_L60 7 d	3.30 ± 0.001	123.4 ± 0.014	122.5 ± 0.015	Mix_C60 7 d	0.27 ± 0.019	20.0 ± 0.019	261.7 ± 0.017
Mix_L60 28 d	0.01 ± 0.016	121.5 ± 0.009	100.9 ± 0.005	Mix_C60 28 d	0.85 ± 0.019	18.9 ± 0.016	250.3 ± 0.018
As(III)_L70 7 d	1.39 ± 0.009	110.1 ± 0.002	75.8 ± 0.004	As(III)_C70 7 d	2.20 ± 0.014	25.1 ± 0.019	1942.6 ± 0.012
As(III)_L70 28 d	1.51 ± 0.016	105.3 ± 0.001	82.4 ± 0.011	As(III)_C70 28 d	1.43 ± 0.005	20.1 ± 0.003	852.1 ± 0.002
As(V)_L70 7 d	3.63 ± 0.015	126.7 ± 0.018	140.9 ± 0.016	As(V)_C70 7 d	1.77 ± 0.020	21.7 ± 0.007	1435.1 ± 0.018
As(V)_L70 28 d	1.36 ± 0.016	123.6 ± 0.009	142.6 ± 0.018	As(V)_C70 28 d	1.38 ± 0.014	15.4 ± 0.018	744.1 ± 0.002
Mix_L70 7 d	2.41 ± 0.007	112.9 ± 0.010	95.4 ± 0.016	Mix_C70 7 d	1.37 ± 0.008	27.2 ± 0.007	1207.4 ± 0.009
Mix_L70 28 d	1.29 ± 0.003	100.8 ± 0.015	102.3 ± 0.019	Mix_C70 28 d	1.44 ± 0.009	19.3 ± 0.002	826.4 ± 0.006

## Data Availability

The data presented in this study are available on request from the corresponding author. The data are not publicly available due to intellectual property agreement.
